# Internet Use and Depressive Symptoms Among Older Adults in China

**DOI:** 10.3389/fpsyt.2021.739085

**Published:** 2021-12-07

**Authors:** Hua-lei Yang, Shuo Zhang, Si-qing Zhang, Lin Xie, Yuan-yang Wu, Yi-dan Yao, Li-li Tang, Zhi-yun Li

**Affiliations:** ^1^School of Public Administration, Zhongnan University of Economics and Law, Wuhan, China; ^2^Department of Population and Labor Economics, University of Chinese Academy of Social Science, Beijing, China; ^3^College of Chemistry and Chemical Engineering, Yantai University, Yantai, China; ^4^College of Politics and Public Administration, Qingdao University, Qingdao, China

**Keywords:** Internet use, depressive symptoms, older adults, digital age, China

## Abstract

This study investigated the effect of using the Internet on depression symptoms of older Chinese, based on 7,801 adults aged over 60 years from the 2018 China Family Panel Studies. Results showed that the elderly who used the Internet reported lower depression scores, and the more frequent they use Internet, the lower their depression scores. Moreover, using the Internet for social contact and entertainment decreased the depression scores of the older adults, but when using Internet for learn, work, and commercial activity, the relief of depressive symptoms disappeared. Therelief of depression symptoms through Internet use were heterogeneous among different groups: the elderly aged 60–70, women, rural residents, and those with lower education attainment. Moreover, Internet use decreased the depression scores by increasing the frequency of contact with their children and increasing the importance of their enjoyment of life. According to the relief of depression by using Internet reasonably, policies should be designed to ensure that all ages could have easy access to the Internet.

## Introduction

Over the past decade, China's population has been aging rapidly. The number of older people aged 60 and over in China climbed to 264 million in December 2020, accounting for about 18.7% of the total population. With the continuous improvement of life expectancy and the increase in the number of elderly people, the mental health of the older people has also received attention. Depression was one of the most common mental illnesses among the elderly, and it is a recurring mental illness. The elderly often suffered the severe depression, and the prevalence of depression that does not meet the clinical diagnostic threshold was quite large in China, especially in the rural areas (1). Depression symptoms played a crucial role in the onset of dementia and other health problems of older people in later life. Thus, more attention should be paid to the depressive symptoms of the elderly.

After 2010, with the popularityof smartphones, the utilization rate of the Internet in China has increased year by year. According to the latest Statistical Report on Internet Development in China, the number of Internet users in China reached 989 million, and the penetration rate reached 70.4% in December 2020 (2). Additionally, since the COVID-19 epidemic, the Internet has brought many conveniences to life and the utilization rate of the Internet has increased sharply during the epidemic. However, due to the low level of education attainment, the decline of cognition and learning, and the difficulty in changing thinking concepts, the proportion of the older people using the Internet was low and was only 11.2% of Internet users are over the age of 60 (2). In addition, some scholars found that Internet use can contribute to mental health by increasing self-efficacy and enriching life experiences to a certain extent, as a way of reducing stress and depression levels (3). Thus, under China's context, was Internet use conducive to the mental health of older people? did using Internet promote the health level of the Chinese older people? The answer to these questions affected the formulation of Internet use policies, especially for promoting the use of the Internet among older people. Therefore, this paper explored the relationship and mechanism between Internet use and the depressive status of older people in China.

Scholars extensively researched and explored the relationship between Internet use and mental health. However, they have not reached a consistent conclusion. Some scholars believed that Internet use reduced the level of depression and was conducive to mental health. In terms of research results, Internet use enhanced people's contact with the outside world, expanded the scope of communication, increased social support, avoided social isolation, and had a positive effect on people's mental health (4–7). Trocchia and Janda (8) made a preliminary attempt to study the differences between elderly Internet users and non-users. After 12 in-depth interviews, they pointed out that the Internet had a significant happiness incentive effect on the elderly. This conclusion coincided with other scholars (9–11). Cotten et al. (12) used the Propensity Score Matching method to study the relationship between Internet use and depression in retired Americans aged 50 and over, and found that Internet use made a positive contribution to the mental health of retired older people. Shillair et al. (13) found using Internet can maintain and enrich older people's social relationships, reduce the effects of loneliness and increase life satisfaction. Confortin et al. (14) noted that the Internet can be seen as a space for older people to enjoy various information, particularly health-related information. Benvenuti et al. (15) selected 271 Italian older adults (aged 60 and over) to study and found that when the older adults used Internet, they would get positive perceptions of online social support, making them to stay closer to family and friends. For older adults with difficulties in mobility, Mcmellon and Schiffman (16) found that using the Internet would expand their social networks and maintain family ties. In addition, Cotten et al. (17) also found that older people who were unwilling to participate in social activities would also benefit from the use of the Internet.

In terms of impact mechanism, William (6) studied the relationship between technology use for social reasons and physical and mental health of older people. Through the use of five technology-based behaviors such as using e-mail, social networking sites, online video, instant messaging and using a smartphone, he found that social technology was related to higher subjective well-being and fewer depressive symptoms, and the associations were mediated by reducing loneliness. Du and Wang (18) used binary logistic regression and Propensity Score Matching to find that Internet use significantly improved the life satisfaction of Chinese older adults, in which community participation played a positive role. Cotten et al. (17) found that using the Internet could help older people overcome the obstacles of time and space to increase the frequency of social interaction, which was conducive to their mental health. Some older people maintained close contact with relatives and friends through the Internet, thus enhancing their support from family and friends, and improving their mental health (16, 19). Heo et al. (20) conducted a study using the data of the Health and Retirement Study in the United States in 2008 and found that Internet use reduced the loneliness of older people by improving social support. Another cross-sectional study also reported that using Internet was associated with greater social support, relief of loneliness, better life satisfaction and better psychological well-being (21). A Korean study (22) also confirmed the mediating role of social support, suggesting that social media use, such as the Internet, indirectly affected quality of life through social support, which in turn benefited their psychological well-being. Older adults who used online social networking sites reported that the strong sense of support from friends and the strong social connection (23). In addition, Lu and Wang (24) found that the information search and dissemination function of the Internet were the main mechanism to improve subjective welfare.

Some scholars held different views that Internet use may promote the emergence of depressive symptoms and have an adversely impact on mental health. Unhealthy Internet behavior reduced communication in real life. When a lot of time was spent on weak ties in the network, it would reduce the time spent on strong ties, destroying real relationships and negatively impacting life satisfaction. “Internet time substitution hypothesis” proposed that Internet use would occupy the time of social communication, reduce opportunities for face-to-face communication, and increase sense of isolation between people (25, 26), which was not conducive to emotional expression and maintenance of social relations, thus increasing inner loneliness and loss (27, 28). Hage et al. (29) tried to explore the relationship between online communication and social connectivity among the elderly, taking 302 older people as the research object, and found that Internet use would reduce real social participation, weakening sense of community belonging. Matsuba (30), Stepanikova et al. (31), Odaci and Elik (32), and Kitazawa et al. (33) found that excessive use of the Internet would make people addicted, produce a sense of loneliness. Elgan (34) also stated that social network addiction became a serious problem. When interacting excessively on social networks, users may experience depression and anxiety (35), because constant social comparisons and invasion of privacy leaded to negative emotions (36).

In 2020, the number of Internet applications in China grew steadily, which has affected people's daily life to a certain extent. Among them, short videos, online payment and online shopping increased most significantly, with growth rates of 12.9, 11.2, and 10.2%, respectively (2). When paying attention to the relationship between Internet and mental health, people were affected differently for different types of Internet using. Lifshitz et al. (37) found that when online social activities, instant messaging and search engines were regarded as part of leisure functions, they were related to participants' well-being, but when they were regarded as instrumental purposes, they did not show such contribution. Other scholars also pointed out that essential Internet use did not improve people's mental health, but showed higher levels of depression and anxiety (38), such as online payment and Internet financial management among the elderly. However, some scholars also pointed out that online games, short videos and other leisure methods provided by the Internet enriched people's lives (39), and social software such as Wechat, Microblog and QQ improved the level of social interaction (9, 40). Search engines could enable them to obtain information related to their hobbies and current events (4). Online platforms such as online shopping and online takeout diversified consumption patterns, which would affect people's mental health, mainly reflected in the improvement of depression.

From the above literature review, few studies focused on the impact of Internet use on depression symptoms and other mental health of Chinese older people, especially in China's context. Contrary to developed countries, the development and popularity of the Internet in China was much later. However, the current digital economy was developing faster. Therefore, the digital gap between urban and rural areas or between older people and young people in China was huge. This paper was based on the data from 2018 China Family Panel Studies (CFPS), which was representative of the current situation of Chinese social families. Most samples in the China Health and Retirement Longitudinal Study (CHARLS) were rural samples, while the samples of CFPS were balanced in urban and rural distribution. The heterogeneity of the Chinese older people group was rarely considered in the existing studies on the impact of depression symptoms and other mental health of older people. There were obvious dissimilarities in urban and rural residents, which included gender differences and education attainment. Therefore, it was necessary to analyze the impact of Internet use on depression symptoms among different population. This paper also discussed the mechanism of Internet use to further enrich the research conclusions. Further, most studies rarely considered the endogenous problem caused by self-selection when investigating the impact of Internet use on depression. Thus, the instrumental variable methods was employed to solve the endogenous problem.

## Materials and Methods

### Study Population and Data Collection

2018 CFPS was used to investigate the effect of Internet use on older adults' depression symptoms. CFPS was a nationally representative and longitudinal survey conducted by the Institute of Social Science Survey of Peking University, starting in 2010. A multi-stage probability proportional to size strategy with implicit stratification was performed in the sampling process. It comprised three stages: County-level as the primary sampling unit, a community, or village for the second stage sampling unit, and the household as the final sampling unit. The CFPS survey consisted of a rich set of socioeconomic questions and information at the levels of children, adults, families, and communities (41). Human participants were approved by Peking University Biomedical Ethics Committee (IRB00001052-14010). Moreover, at the beginning of the survey, the questionnaire included the statement that the collected data would be used for scientific research. Written informed consent was obtained from each participant. For our analytic file, we used claims data from CFPS 2018, which were the latest survey data available at the time of the study. The CFPS 2018 survey collected 44,000 personal questionnaires, 22% of which was completed by telephone interviews. Given that this article studied the depression symptoms effects of the Internet use on the elderly, data including people who were under 60 years in 2018 were excluded from the study.

Depression symptoms was measured by a depression score of an eight-item version of the Center for Epidemiologic Studies (CES-D) scale in the CFPS 2018. The CES-D scale was a commonly used measure of depression symptoms in older adults (42–45) and was the only measure of depression symptoms in the CFPS. The CES-D scale consisted of eight questions about the feel of last week, namely, question N406 “I feel depressed,” question N407 “I feel it takes a lot of effort to do anything,” question N411 “My sleep is not good,” question N412 “I feel happy,” question N414 “I feel lonely,” question N416 “I live happily,” question N418 “I feel sad,” and question N420 “I feel that life can't go on.” The four options of the eight questions were hardly (<1 day), sometimes (1–2 days), often (3–4 days), most of the time (5–7 days). They were assigned to 1, 2, 3 and 4, respectively. Among them, because of the fourth (I feel happy) and the sixth question (“I live happily),” which reflected positive emotions, the two questions were processed in reverse order, resulting in a depression score of 8–32. Higher scores indicated greater severity of depression symptoms.

We divided the study population into two groups: Internet users and non-users, according to the answers of the two questions, namely, question U201 “Do you use mobile Internet?”, and question U202 “Do you use computer Internet?.” The respondent was assigned to the Internet users group if they used mobile Internet or computer Internet in 2018 and the Internet non-users group if they did not surf the Internet in 2018. Moreover, we used the frequency of Internet use to replace the independent variable to further investigate the depression symptoms effect of Internet use. According to answers of question U701 “Frequency/times of using the Internet for learning,” question U702 “Frequency/times of using the Internet for work,” question U703 “Frequency of using the Internet for social interaction (times),” question U704 “Frequency/times of using the Internet for entertainment,” and question U705 “Frequency/times of using the Internet for commercial activity, such as using online banking, online shopping.” The highest frequency among them was chosen as the frequency of using the Internet, and the final assignment was 0 to 6, which means “not used,” “once a few months,” “once a month,” “2–3 times a month,” “1–2 times a week,” “3–4 times a week,” and “almost every day,” respectively. Moreover, we examined the impact of different kind of the Internet usage on the depressive symptoms of the old adults. These variables were dummy variables formed according to questions U701, U702, U703, U704, and U705.

Internet use increased the social connections of the elderly and simultaneously allowed the elderly to have more access to the colorful world. However, due to data limitations, we only examined the frequency of contact with children in social connections and the emphasis on the joy of life. The frequency of contact with children was based on the average contact frequency of each child in question F306 “In the past 6 months, how often did you contact your child by phone, SMS, letter or email?.” The value was 0–6 and the larger the value, the more times they contact. The joy of life value was obtained by answering the question M503 “the importance of having fun in life,” with a value of 1–5.1 meaned not important, and five meaned very important.

However, other factors may generate bias when estimating the results. We collected the following characteristics for the study participants as covariates: age (continuous variable); sex (1 = male; 0 = female), education (0 = illiteracy, 1 = high school or lower, 2 = college or higher); Hukou (1 = non-farm; 0 = farm); marriage (1 = married/in de facto relationship, 0 = other); residence (1 = urban, 0 = rural); log of household saving per capita (lnfinc); and its square (Infinc2). These variables were selected because (1) they are highly related to the occurrence of depression symptoms and other mental problems, and (2) they are obtainable from the claims data directly or through the working definition.

### Main Empirical Strategy

The ordinary least squares estimation and ordered Probit estimation were employed first within this study.


(1) $$\begin{array}{r} Depression_{i}=\alpha_{1}+\beta_{1}Internet_{i}+\gamma_{1}Z_{i}+\varepsilon_{i} \end{array}$$

Here, the subscript *i* referred to the individual. *Depression*_*i*_ was the dependent variable related to the outcomes of depression scores. For the main independent variables, *Internet*_*i*_ was a dummy variable equal to one for the Internet usage experimental group and zero for the non-usage control group; and vector *Z*_*i*_ was the covariates.

In ordinary least squares estimation, *Depression* was treated as continuous data. In the ordered probit (Oprobit) model, *Depression* was treated as a ranking variable, and it was necessary to deduce the estimator by using latent variables high likelihood estimate method.

### Robustness Analysis: Impact of Frequency of Internet Usage

If the relation between Internet use and depression symptoms of older adults was robust, the different indicators to measure Internet use would have a same impact on the estimation results. Thus, the frequency of Internet use was taken as a new independent variable, and the following model was constructed to test the robustness of Internet use on depression of older adults.


(2) $$\begin{array}{r} Depression_{i}=\alpha_{1}+\beta_{1}Frequency_{i}+\gamma_{1}Z_{i}+\varepsilon_{i} \end{array}$$

Here, *Frequency*_*i*_ was the frequency of individual use of the Internet, and the meaning of other variables was consistent with Equation (1).

### Endogenous Problem

However, endogenous factors might bias our main results. That is, the decision to use the Internet, together with the individual's depression symptoms, may be determined or influenced by some missing or unobserved variables. Additionally, mental health status affected a person's behavior, which, in turn, may affect an individual's online behavior, creating reverse cause-and-effect problems. To overcome these problems and check the robustness of the main results, we first employed the two-stage least squares method (2SLS).


(3) $$\begin{array}{r} Internet_{i}=\varphi_{1}+\phi_{1}X_{i}+\lambda_{1}Z_{i}+\varepsilon_{1i} \end{array}$$

(4) $$\begin{array}{r} Depression_{i}=\varphi_{2}+\phi_{2}{\bar{Internet}}_{i}+\lambda_{2}Z_{i}+\varepsilon_{2i} \end{array}$$

Here, *X*_*i*_ was the instrumental variable; ${\bar{Internet}}_{i}$ was the fitted value of Equation (3).

Considering that the participants' depression scores and Internet use in this study were both discrete variables, methods based on continuous variables may be ineffective (46). Therefore, we employed the conditional mixed process method (CMP) proposed by Roodman (47) to re-estimate the model.

The CMP also belonged to the two-stage regression. As in the 2SLS model, the first stage of the CMP method was to find the instrumental variables of the main independent variable and evaluate their correlation. In the second stage, the instrumental variables were substituted into the model for regression, and the exogeneity of Internet use was verified according to the endogeneity test parameters. If the endogeneity test parameters were significantly different from 0, it indicated that the model has endogeneity problems—the estimation results of the CMP method were superior to Oprobit model. Conversely, if the endogeneity test parameters were not significantly different from 0, the Oprobit model estimation results can be referred to. The CMP method adopted simultaneous likelihood estimation in the second stage. In this study, the Internet penetration rate of the county level was taken as the instrumental variable of whether participants used the Internet, and the 2SLS and CMP methods were used, respectively.

### Mediating Effect Model

This article referred to the intermediary effect test procedure proposed by Baron and Kenny (48) to explore whether the Internet use of the elderly can improve the depression status by increasing the frequency of social contact and increasing the joy of life.


(5) $$\begin{array}{r} Depression_{i}=\alpha_{1}+\beta_{1}Internet_{i}+\gamma_{1}Z_{i}+\varepsilon_{i} \end{array}$$

(6) $$\begin{array}{r} M_{i}=\alpha_{2}+\beta_{2}Internet_{i}+\gamma_{2}Z_{i}+\varepsilon_{\text{i}} \end{array}$$

(7) $$\begin{array}{r} Depression_{i}=\alpha_{3}+\beta_{3}Internet_{i}+\delta M_{i}+\gamma_{3}Z_{i}+\varepsilon_{\text{i}} \end{array}$$

Here, *M*_*i*_ was the intermediary variable. The coefficient β_1_ in Equation (1) represented the total effect of the elderly's use of the Internet on depression. The coefficient β_2_ in Equation (5) was the effect of Internet use on the intermediary variable. The coefficient δ in Equation (6) indicated that the effect of the intermediary variables *M*_*i*_ on depression after controlling Internet usage, and the coefficient β_3_ represented the direct effect of Internet use on depression under the control of the intermediary variables *M*_*i*_. At this time, the mediation effect was equal to the indirect effect, that is, the product of coefficients β_2_δ.

The ideas of mechanism analysis were as follows: First, equation (1) was estimated, and on the basis of significant of β_1_, equations (5) and (6) were further estimated, among which *M*_*i*_ were the mediating factors concerned in this paper. If both β_2_ and δ were significant, then the mediating effect was significant. If at least one of them was not significant, further Sobel test should be conducted for the cross term β_2_δ. If the Sobel Z statistic was significant, then the mediating effect exists. If not, the indirect effect was not significant.

## Results

### Descriptive Statistics

Table 1 provided the descriptive statistics of the sample. In the full sample, participants had an average self-reported depression score of 13.818 (out of 32, s.d. = 4.525), and about 12.7% of respondents used mobile devices or computers to access the Internet. The sample had slightly more males (50.4%) than females, with the average age of participants being 68 years old. Most of the participants had lower than high school education (97.5%). About 48.4% of participants lived in urban areas. People registering in farm (30.1%) were considerably less numerous than non-farm. Most participants were married or in de facto relationships (81.2%).

**Table 1:** Descriptive statistics for the sample.

	**Full sample**			**Internet users**			**Internet non-users**		
	***N***	**Mean**	**sd**	***N***	**Mean**	**sd**	***N***	**Mean**	**sd**
Depression	7,801	13.818	4.525	988	12.408	3.726	6,813	14.023	4.594
Internet	7,801	0.127	0.333	988	1	0	6,813	0	0
Frequency	7,801	0.624	1.774	988	4.924	1.917	6,813	0	0
Learn	7,800	0.044	0.206	988	0.352	0.478	_	_	_
Work	7,800	0.01	0.098	988	0.076	0.265	_	_	_
Social contact	7,801	0.093	0.29	988	0.734	0.442	_	_	_
Entertainment	7,800	0.093	0.291	988	0.736	0.441	_	_	_
Commercial activity	7,800	0.03	0.171	988	0.237	0.426	_	_	_
Age	7,801	68.192	6.325	988	65.925	5.066	6,813	68.521	6.421
Gender	7,801	0.504	0.5	988	0.603	0.489	6,813	0.49	0.5
Education	7,715	0.669	0.608	974	1.146	0.715	6,741	0.6	0.558
Illiteracy	7,715	0.38	0.485	974	0.08	0.272	6,741	0.424	0.494
High school and less	7,715	0.595	0.491	974	0.807	0.395	6,741	0.565	0.496
College and above	7,715	0.025	0.155	974	0.113	0.317	6,741	0.012	0.108
Hukou	7,792	0.301	0.459	986	0.693	0.462	6,806	0.244	0.43
Marriage	7,801	0.812	0.391	988	0.888	0.316	6,813	0.801	0.399
Residence	7,756	0.484	0.5	981	0.782	0.413	6,775	0.441	0.497
lnfinc	7,754	9.413	1.233	981	10.284	1.124	6,773	9.287	1.197
lnfinc2	7,754	90.127	21.804	981	107.013	21.196	6,773	87.682	20.784
The child contact frequency	7,212	4.271	0.688	919	4.393	0.67	6,293	4.253	0.689
The importance of having fun in life	7,789	4.166	0.986	988	4.303	0.823	6,801	4.146	1.007

Table 1 also provided the mean of variables for Internet users and the non-users groups. Among the Internet users, 35.2% of them used the internet for learning, 7.6% for work, 73.4% for social contact, 73.6% for entertainment, 23.7% for commercial activities. The results also showed that older persons who used the Internet were more likely to have lower self-reported depression scores (mean = 12.408, s.d. = 3.726) than the non-users group (mean = 14.023, s.d. = 4.594). Moreover, compared to the non-users group, participants who used the Internet were more likely to be younger (mean = 66, s.d. = 5.066), be male (60.3%), higherly educated (mean = 1.146, s.d. = 0.715), registering in non-farm (69.3%), married (88.8%), in an urban area (78.2%), and having more household savings (mean = 10.3, s.d. = 1.124).

### The Relationship Between Internet Usage and the Depressive Symptoms of Older Adults

The ordinary least squares (OLS) and Ordered Probit regression results were presented in Table 2. Columns (1) and (2) showed the results of the OLS method. The difference in column (2) showed the result of controlling all other variables. Columns (3) and (4) showed the results of the Ordered Probit method, and column (4) controlled all other variables. Column (2), in Table 2, showed that participants who used the Internet compared with Internet non-welfare residents had significantly lower depression scores by 0.3053 (*p* < 0.05). Moreover, the results in all models showed negative coefficients and were statistically significant at the 5% level, suggesting that Internet use reduced the depressive symptoms of older persons. However, the estimated effect might still be biased because Internet use was likely to be selected and endogenous with depression. These findings should be viewed alongside further robustness checks, as described below.

**Table 2:** Results of the ordinary least square and ordered probit methods.

	**OLS**		**Ordered Probit**	
	**(1)**	**(2)**	**(3)**	**(4)**
Internet	−1.6150^***^	−0.3053^**^	−0.3723^***^	−0.0750^**^
	(0.0000)	(0.0345)	(0.0000)	(0.0474)
Age		−0.0122		−0.0037^*^
		(0.1572)		(0.0681)
Gender		−1.1232^***^		−0.2829^***^
		(0.0000)		(0.0000)
Education		−0.3965^***^		−0.0891^***^
		(0.0003)		(0.0006)
Hukou		−0.2310		−0.0718^**^
		(0.1112)		(0.0423)
Marriage		−1.6656^***^		−0.3725^***^
		(0.0000)		(0.0000)
Residence		−0.4855^***^		−0.1231^***^
		(0.0001)		(0.0000)
ln*finc*		0.1687		0.0515
		(0.4844)		(0.3104)
ln*finc*^2^		−0.0425^***^		−0.0106^***^
		(0.0018)		(0.0004)
_cons	14.0229^***^	20.4235^***^		
	(0.0000)	(0.0000)		
Province dummies	No	Yes	No	Yes
*N*	7,801	7,617	7,801	7,617
r2_a	0.0140	0.1304		

***, **, and * *indicate significance at 1, 5, and 10%, respectively. Standard errors are presented in parentheses*.

Apart from using the Internet, several sociodemographic variables affected older adults' depression. We found that men tend to have lower depression scores than women (*r* = 1.123, *p* < 0.01). However, age had no statistically significant effect on mental health. People with a higher level of education reported lower depression scores. The elderly who were non-farm were more likely to have lower depression scores. Furthermore, we found a statistically significant reduction in depression scores for married and de facto relationships and those who live in urban areas. Finally, the association between household savings and depression was found to be statistically significantly positive.

### Impact of Frequency and Content of Internet Usage on the Depressive Symptoms of Older Adults

Regression results of the impact of frequency and content of Internet usage on older adults' depressive symptoms were reported in Table 3. All the results were estimated by OLS. As shown in column (1), the average effect of more frequent Internet use can statistically significantly reduced the depression score by 0.0573 (*p* < 0.05), suggesting that the more frequently older persons use the Internet, the lower their depression scores. As shown in columns (2) to (6) different types of Internet usage have different effects on older people. Using the Internet for social contact and entertainment statistically significantly reduced the depression scores of the older adults, namely, using the Internet for social contact reduced the depression score by 0.2586 (*p* < 0.1), using the Internet for entertainment reduced the depression score by 0.4106 (*p* < 0.01). However, using the Internet to learn, work, and business activities had no statistically significant effects.

**Table 3:** Impact of internet use frequency and the kind of internet usage using OLS.

	**(1)**	**(2)**	**(3)**	**(4)**	**(5)**	**(6)**
Frequency	−0.0573^**^	−0.0134				
	(0.0293)	(0.9479)				
Learn		−0.0134				
		(0.9479)				
Work			−0.1094			
			(0.7938)			
Social contact				−0.2586^*^		
				(0.0974)		
Entertainment					−0.4106^***^	
					(0.0080)	
Commercial activity						−0.0532
						(0.8274)
Age	−0.0122	−0.0099	−0.0104	−0.0115	−0.0125	−0.0104
	(0.1573)	(0.2451)	(0.2235)	(0.1824)	(0.1455)	(0.2229)
Gender	−1.1251^***^	−1.1282^***^	−1.1248^***^	−1.1301^***^	−1.1239^***^	−1.1255^***^
	(0.0000)	(0.0000)	(0.0000)	(0.0000)	(0.0000)	(0.0000)
Education	−0.3966^***^	−0.4253^***^	−0.4243^***^	−0.4043^***^	−0.3953^***^	−0.4237^***^
	(0.0003)	(0.0001)	(0.0001)	(0.0002)	(0.0003)	(0.0001)
Hukou	−0.2314	−0.2669^*^	−0.2679^*^	−0.2432^*^	−0.2244	−0.2664^*^
	(0.1092)	(0.0638)	(0.0618)	(0.0923)	(0.1205)	(0.0638)
Marriage	−1.6669^***^	−1.6644^***^	−1.6690^***^	−1.6654^***^	−1.6678^***^	−1.6690^***^
	(0.0000)	(0.0000)	(0.0000)	(0.0000)	(0.0000)	(0.0000)
Residence	−0.4867^***^	−0.4972^***^	−0.4964^***^	−0.4912^***^	−0.4851^***^	−0.4958^***^
	(0.0000)	(0.0000)	(0.0000)	(0.0000)	(0.0001)	(0.0000)
ln*finc*	0.1711	0.1945	0.1935	0.1781	0.1709	0.1935
	(0.4802)	(0.4203)	(0.4221)	(0.4618)	(0.4806)	(0.4225)
(ln*finc*)^2^	−0.0426^***^	−0.0445^***^	−0.0444^***^	−0.0432^***^	−0.0425^***^	−0.0444^***^
	(0.0019)	(0.0011)	(0.0011)	(0.0016)	(0.0019)	(0.0011)
_cons	20.4054^***^	20.1238^***^	20.1503^***^	20.3096^***^	20.4425^***^	20.1623^***^
	(0.0000)	(0.0000)	(0.0000)	(0.0000)	(0.0000)	(0.0000)
Province dummies	Yes	Yes	Yes	Yes	Yes	Yes
*N*	7,617	7,617	7,616	7,617	7,616	7,616
r2_a	0.1304	0.1300	0.1297	0.1302	0.1305	0.1297

***, **, and * *indicate significance at 1, 5, and 10%, respectively. Standard errors are presented in parentheses*.

### Endogenous Problems

The 2SLS and CMP methods were employed to address potential endogeneity problems. The Internet penetration rate of the county was used as an instrumental variable for the individual's Internet usage. On the one hand, the laying of network infrastructure always has regional characteristics, and personal Internet usage tended to be related to the Internet penetration rate in counties. On the other hand, whether the network infrastructure was laid in a certain area was greatly affected by the economic level and environment of the area. Therefore, the Internet penetration rate was not related to residents' depression at the micro-individual level. We used the 2SLS and CMP methods in a two-stage process. First, we estimated the effect of the Internet penetration rate at the county level on Internet use. Second, we estimated the effect of Internet use on depression by regression (47). The estimation results were reported in Table 4.

**Table 4:** Results Estimated by the 2SLS and CMP Methods.

	**2SLS**		**CMP**	
	**Stage I**	**Stage II**	**Stage I**	**Stage II**
Internet		−2.9012^***^		−0.3774^***^
		(1.0356)		(0.1139)
Internet penetration rate of the	0.4584^***^		3.9455^***^	
county level	(0.0367)		(0.1557)	
DWH	6.4938			
atanhrho_12			0.1745^***^	
			(0.0632)	
Covariates	Yes	Yes	No	Yes
Province dummies	Yes	Yes	No	Yes
N	7,617		7,617	

***, **, and * *indicate significance at 1, 5, and 10%, respectively. Standard errors are presented in parentheses. DWH is the estimated parameter of Durbin–Wu–Hausman test. The atanhro_12 is a parameter in the CMP model. If it is different from zero, then there are unobserved factors simultaneously affecting the dependent variable and main independent variable, suggesting the main independent variable is endogenous*.

As shown in Table 4, in the first stage regression of the 2SLS model and the CMP method, the Internet penetration rate of the county was statistically significantly positively correlated with Internet usage, meeting the dependency condition of the instrumental variables. Moreover, the endogeneity test parameter atanhrho_12 in the CMP estimation was statistically significant, also indicating that Internet usage was an endogenous independent variable.

Next, the second stage regression results of the 2SLS model showed that Internet usage still had a statistically significant and reduced effect on the depression symptoms of participants after correcting for possible endogenous bias and was statistically significant at the 1% level, suggesting a very reliable causality. The results from the CMP estimation were still negative but much less so. This further confirmed the reduced effect of Internet usage on the depression symptoms of the participants. Compared to other results in this study, 2SLS point estimates showed larger impacts of Internet use on depression symptoms, while the sign of impacts was unchanged. One possible reason was that the results of the 2SLS estimations were likely to be inconsistent when the regression model was specified as a linear form, but the dependent variable was a count or dichotomous variable. More generally, 2SLS estimates may be inconsistent because they reflected the linear local average treatment effect (LATE), not the average treatment effect (ATE) for the whole population. A Durbin–Wu–Hausman test was used to check the model's endogeneity, and the null hypothesis that all explanatory variables were exogenous cannot be strongly rejected (6.4938 [p < 0.01]). The endogeneity problem caused no statistically significant estimation bias in our analysis, and the main results were reliable. Overall, all regressions produced similar results, although the magnitudes and significances appeared to be slightly different.

### Effects by Population Groups

Given that the impact of Internet usage on mental health was likely to have differential effects in terms of individual characteristics, the whole sample was divided into subsamples according to different criteria, with two subsamples based on age (under 70 years old, or above), two subsamples based on sex (male or female), two subsamples based on residence (urban or rural), and three subsamples based on education level (illiteracy, high school and below, or college and higher). The CMP was conducted separately for these subgroups. Table 5 displayed the estimates of the population groups.

**Table 5:** Results of the subgroups analysis (age, sex, residence).

**Variables**	**Age**		**Sex**		**Residence**	
	**<70**	**> =70**	**Male**	**Female**	**Urban**	**Rural**
Internet	−0.2938^*^	−0.1627	−0.2090	−0.4455^**^	−0.3782^**^	−0.7812^***^
	(0.1624)	(0.2484)	(0.1977)	(0.1761)	(0.1589)	(0.2205)
atanhrho_12	0.1328	0.1031	0.1161	0.2035^**^	0.1826^*^	0.3981^***^
	(0.0937)	(0.1372)	(0.1152)	(0.0994)	(0.0961)	(0.1063)
Results of First Stage—use Internet or not						
Internet penetration rate of the county level	2.5408^***^	2.5749^***^	2.5479^***^	2.5195^***^	2.6401^***^	1.8944^***^
	(0.2214)	(0.3691)	(0.2524)	(0.2878)	(0.2287)	(0.3598)
Covariates	Yes	Yes	Yes	Yes	Yes	Yes
N	4,921	2,696	3,839	3,778	3,678	3,939
**Variables**		**Education**				
**Results of the subgroups analysis (education)**						
		Illiteracy	High school or less	College or more		
Internet		−0.7993^*^	−0.3952^**^	−0.6619		
	(0.4207)	(0.1554)	(0.5143)		
atanhrho_12		0.3878^**^	0.1822^**^	0.4735		
		(0.1814)	(0.0904)	(0.3051)		
Results of First Stage—use Internet or not						
Internet penetration rate of the county level		1.8105^***^	2.5785^***^	2.5558^***^		
		(0.5212)	(0.2131)	(0.9065)		
Covariates		Yes	Yes	Yes		
N		2,906	4,524	187		

***, **, and * *indicate significance at 1, 5, and 10%, respectively. Standard errors are presented in parentheses*.

As shown in Table 5, Internet usage had a heterogeneous effect on mental health among older people based on age group. Among those aged 70 or below, there was a statistically significantly lower depression score (*r* = −0.2938, *p* < 0.1). Conversely, there were no statistically significant effects for participants above 70 years, and the effects were much smaller (*r* = −0.1627, *p* > 0.1). In terms of sex, Internet use had a statistically significant negative impact on self-reported depression scores for women (*r* = −0.4455, *p* < 0.05) but not for men (*r* = −0.2090, *p* > 0.1). The positive effects of Internet usage on mental health vary by residential area. Participants in rural areas showed statistically significant lower depression scores if they used the Internet. However, for those in urban areas, the effect was much smaller.

The effects of Internet usage on depressive symptoms of old adults varied according to educational level. The effect of Internet usage on depressive symptoms was only statistically significant in the middle and low education level groups. Self-reported depression scores of older people with an education level of high school or below were lower for the Internet usage group than for the non-usage group. Moreover, the effect of Internet usage on depressive symptoms was much larger for illiterate participants. However, for those with a college education or higher, the coefficients of CMP estimation were statistically insignificant, indicating that the depressive symptoms of those with higher education would not be affectedif they used the Internet.

### Intermediary Mechanism

The aforementioned literature analysis found that the effect of Internet use on depression is mainly through two aspects: first, Internet use increased the connection between older adults and society, increasing social support. Second, increased entertainment can be obtained using the Internet, which would enrich the lives of older adults, reducing the depression of the elderly. Therefore, we selected the average frequency of contact with children and the self-rated importance of life pleasure as the mediating variables to analyse the mechanism of the impact of Internet use on the depressive symptoms of the elderly.

Results were reported in Table 6. Models 1 and 3 were the regression results of the intermediary variables used by the Internet, and models 2 and 4 were the regression results of the benchmark model after adding the intermediary variables. As shown in Table 6, Internet use statistically significantly increased the frequency of children's contact (*r* = 0.5565, *p* < 0.01), increased the importance of the elderly's joy in life (*r* = 0.0772, *p* < 0.05), and statistically significantly reduced the depression scores of the older people through the two.

**Table 6:** Mediating effect of children contact frequency and life fun.

	**(1)**	**(2)**	**(3)**	**(4)**
	**Contact frequency**	**Depression**	**Life fun**	**Depression**
Internet	0.5565^***^	−0.1843	0.0772^**^	−0.2386^*^
	(0.0000)	(0.2138)	(0.0205)	(0.0927)
Contact frequency		−0.1372^***^		
		(0.0000)		
Life fun				−0.9273^***^
				(0.0000)
Covariates	Yes	Yes	Yes	Yes
Province dummy	Yes	Yes	Yes	Yes
N	7,127	7,127	7,605	7,605
r2_a	0.1361	0.1372	0.0321	0.1698

***, **, and * *indicate significance at 1, 5, and 10%, respectively. Standard errors are presented in parentheses*.

## Discussion

Most older adults in our sample did not use the Internet (87.3%). Although not being able to use the Internet was currently the norm among the older Chinese, rates of Internet users were rapidly increasing. Younger generations have a much greater chance of being Internet users. Demographics indicated that Internet users had a higher standard of living, tended to be younger, higher educated, registering in non-farm, married, and in urban areas. These results were consistent with previous research highlighting the relationship between socioeconomic status and Internet use (49–51).

Examination of the self-reported depression scores for Internet users and non-users provided support for the hypothesis that using the Internet reduced depressive symptoms older adults. Internet users experienced lower levels of depressive symptoms. These effects were significant after controlling for demographic variables, including economic standards of living, and after overcoming endogenous problems. These results supported previous findings linking Internet use to better health and well-being (4, 17, 52). Moreover, the frequency and kinds of Internet usage had an important impact on older adults' depressive symptoms. The more frequently older persons use the Internet, the lower their depression scores. Using the Internet for social contact and entertainment reduced the depression scores of the older adults, while using the Internet to learn, work, and business activities don't.

Furthermore, different impact emerged among Internet users and non-users based on demographic variables. Only the younger group of older adults had statistically significantly lower depression scores. It was relatively easy for the younger group of older adults to use the Internet. Therefore, they had access to various kinds of information through more frequent use and thus maintained a good connection with social affairs. However, older adults above 70 years were more likely to focus on reconciling with themselves and less likely to be interested in the outside world (53, 54). Access to the Internet was more challenging for those older adults (49, 55–60), and their enthusiasm waslacking (61, 62). Moreover, the enhancement of their learning ability and social participation through Internet use was relatively limited. Thus, the benefits of reducing depression were correspondingly limited. Further, the reducing depression effect was more apparent in older women than in older men when using the Internet. This was possibly because women were more likely to be happy from social activities. The Internet made it easier to keep in touch with friends and family. Additionally, lower depression scores were found for the lower education group and the illiterate group who used the Internet, whereas no significant differences were found for the high education level group. The variance may be attributed to the fact that people with higher education levels tend to have more social capital in the first place (63). However, for those with lower education or who for those who were illiterate, the Internet mainly provided a way to explore the world and social interaction.

The relief of depression when using the Internet was more obvious among rural residents than among urban residents. In China, this was because the Internet in urban areas developed earlier and was matured in urban. For urban residents, the Internet may be commonplace. The development and popularization of the Internet started late in rural areas and was now in a period of rapid growth, making it still a novelty. For rural residents, the mass of information, instant social media, and convenience of e-commerce provided a stronger sense of accomplishment and satisfaction compared with urban Internet users. Therefore, it was necessary to further strengthen the construction of Internet infrastructure in rural areas and make Internet use more convenient for older adults.

Furthermore, Internet use reduced the depression scores of the elderly by increasing the frequency of connection with their children and importance of their enjoyment of life. This was consistent with the findings of Cotten et al. (17), Braun (64), and Benvenuti et al. (15). Internet use helped older adults to maintain family ties and obtain more emotional support from family and friends. It also opened the eyes of the older person so that they can learn about and have a more interesting life.

Since depressive symptoms have a great impact on health in old age, we would like to propose relevant interventions measures to reduce depressive symptoms of the older adults (65, 66). Firstly, for older people themselves, it was important to share their interests and enrich their entertainment life to improve their sense of life and well-being (67), which can improve their gastrointestinal, cardiovascular and cerebrovascular discomforts and alleviate somatic discomfort (68). It can also expand the scope of social activities for older people and promote them to face life positively. Secondly, in terms of family and community, which were closely related to the lives of older people, social networks can alleviate depression in older people, especially support from family (69), and children should give older people more companionship. High frequency and quality interactions were beneficial for older people to receive material and spiritual support from family members (70). Community health service and activities for the elderly should be promoted (71). It was also necessary to establish electronic health records for older people and to realize networked management of family and community healthcare institutions, such as health education, medication and psychological counseling (72). Finally, on a social level, good social relationships affected older people's psychological well-being in terms of positive emotions such as a sense of belonging and security (73), encouraging older people to increase their contact with society and actively engage with people to address psychosocial barriers and improve emotional and physical well-being (74), which was consistent with other scholars (75, 76). The risk of depression can be reduced by encouraging older people to take an active part in voluntary activities to enhance their sense of worth and identity (77). In addition, the state should pay attention to older people's job satisfaction while delaying the retirement age, and choose satisfying jobs that take into account their own interests to further reduce the risk of depression.

## Conclusion

In general, older people in China who used the Internet have lower depression scores than those who do not. Demographic differences in Internet use highlighted that policy should pay particular attention to the accessibility of Internet use for the vulnerable group, so as to achieve a healthy China strategy. First, accelerate the construction of Internet infrastructure and further improve the coverage of broadband networks. There is a need to improve the accessibility of Internet use for older people, especially who in rural areas. The development of the Internet in urban and rural areas is not balanced due to the gap of urban-rural development. Therefore, to improve the usage rate of the Internet in rural older people, we should realize the same network and speed in urban and rural areas and accelerate the development of the Internet in rural areas. Second, improve the attitude to Internet of older people, by cultivating their ability, reducing the sense of powerlessness. It is necessary to conduct Internet use skills training to help them become familiar and master the use of various applications, such as health management, leisure, entertainment, and learning. This will eliminate the technical threshold of Internet use, enrich the content of Internet use of older people, and improve their sense of happiness and attainment. Finally, standardize the Internet use content and improve the experience of Internet use of older people. Relevant departments should strengthen the review and supervision of relevant contents during the use of the Internet by older people, use big data to more accurately understand their potential needs. In addition, actively establish a community network environment focusing on entertainment and leisure, social chat and learning, using network technology to improve the online social participation ability and reduce the depression and loneliness of older people.

This study has some limitations. Firstly, the mediating role of social participation of all kinds has not been fully explored. Second, the amount of Internet usage time may also be relevant to the depression outcome (17), but we did not address this specific issue due to data limitation. Moreover, the sampling weights could be used in further analysis. Further examinations of the interrelationships between Internet use and depressive symptoms are encouraged in future researches.

## Data Availability Statement

Publicly available datasets were analyzed in this study. This data can be found at: http://www.isss.pku.edu.cn/cfps.

## Ethics Statement

The studies involving human participants were reviewed and approved by CFPS was undertaken according to the guidelines laid down in the Declaration of Helsinki and all participants signed an informed consent form. Human participants were approved by Peking University Biomedical Ethics Committee (IRB00001052-14010-exemption). The patients/participants provided their written informed consent to participate in this study.

## Author Contributions

SZ and H-lY conceived this research. H-lY, Z-yL, and LX were responsible for the methodology. S-qZ conducted software analyses. SZ and Y-yW conducted necessary validations. L-lT conducted a formal analysis and managed the investigation. SZ and S-qZ gathered resources, curated all data, wrote/prepared the original draft, and were responsible for project administration. LX and Z-yL reviewed, edited the manuscript, and were responsible for visualization. H-lY supervised the project and acquired funding. All authors contributed to the article and approved the submitted version.

## Funding

This work was supported by the Humanities and Social Sciences Fund of the Ministry of Education (Grant Number: 19YJC790167).

## Conflict of Interest

The authors declare that the research was conducted in the absence of any commercial or financial relationships that could be construed as a potential conflict of interest.

## Publisher's Note

All claims expressed in this article are solely those of the authors and do not necessarily represent those of their affiliated organizations, or those of the publisher, the editors and the reviewers. Any product that may be evaluated in this article, or claim that may be made by its manufacturer, is not guaranteed or endorsed by the publisher.
